# Dietary Antioxidants and Health Promotion

**DOI:** 10.3390/antiox7010009

**Published:** 2018-01-12

**Authors:** Dejian Huang

**Affiliations:** Food Science and Technology Programme, c/o Department of Chemistry, 3 Science Drive 3, National University of Singapore, Singapore 117543, Singapore; chmhdj@nus.edu.sg

Accumulating scientific evidence suggests that over-production of reactive oxygen species (ROS) may be the root cause of chronic diseases such as cancer, cardiovascular diseases, neurodegeneration, and ageing *per se* [1,2]. Logically, dietary antioxidants, those that can mitigate the damaging effects of ROS [3], have been suggested to be beneficial for health promotion, although this is a hotly debated topic, as too little ROS activity may have undesirable health consequences [4]. Nonetheless, the research on dietary antioxidants has been developing rapidly. This special issue collects a small sample of 6 articles, most of them mini reviews on dietary antioxidant and health. These articles provide timely updates on the respective topics covered.

Among the plant-based polyphenolic antioxidants, proanthocyanidins (PACs), the oligomers of flavan-3-ol, are the most studied group of phytochemicals. PACs are present in a wide spectrum of plant foods such as berries (i.e., grapes, cranberry, and blueberry), vegetables (e.g., okra) [5]. Due to their structural diversity and complexity, PACs from different fruits and edible plants have different bioactivities. For example, cranberry contains mainly A-type proanthocyanidins, which are believed to be responsible for its health benefits against urinary tract infection [6], while PACs found in okra are potent inhibitors of starch hydrolases [7]. Due to their high phenolic group content, PACs are potent radical scavengers in addition to their structurally specific bioactivities. Cat’s claw (*Uncaria tomentosa* L.) is a traditional medicinal plant in traditional medicine, distributed in South and Central America with anti-inflammatory and antioxidant properties, as well as other health benefits [8]. In the article by Bartolomé and collaborators, polyphenols were extracted from the leaves, stems, bark and wood of *U. tomentosa* harvested from several regions of Costa Rica [9]. It was found that *U. tomentosa* contains mainly B-type proanthocyanidins composed of monomeric units of (epi)catechins, propelargonidins (epi)afzelechin units with degrees of polymerization ranging from 3 to 11. The proanthocyanidin-rich phenolic extracts are good radical scavengers, with ORAC (Oxygen Radical Absorbance Capacity) values between 1.5 and 18.8 mmol TE/g), as well as antimicrobial activity and cytotoxicity against gastric adenocarcinoma AGS and colon adenocarcinoma SW620 cell lines. These findings further enrich our knowledge of proanthocyanidins as bioactive constituents in traditional herbs.

In addition to fruits, vegetables and medicinal plants, spices and culinary herbs have been added to food to enhance flavor and improve their organoleptic properties. The antioxidant components in spices are of great interest. In the mini review by Nemzer and coworkers, the antioxidant compounds that are found in common spices such as ginger, turmeric and garlic, used in our daily cuisine, are summarized [10]. The salient feature of the antioxidants in spices is their structural diversity, with organosulfides being contained in garlic, curcumins in turmeric, carotenoids in saffron, and proanthocyanidins in cinnamon barks. As a result, these spices possess a broad spectrum of health promotion activities. 

Although polyphenolic antioxidants are widespread in the plant kingdom, their concentrations are typically low. Effective extraction and separation of the antioxidant compounds from raw plant tissues are desired to manufacture dietary antioxidants that have sufficient potency when used as dietary supplements or as functional food additives. The chemical diversity of antioxidants, as well as plant tissue composition diversity, demands different extraction methods—chemical, physical, or a combination of both—for the effective extraction of specific plant antioxidants. To this end, Proestos and coworkers demonstrated that by applying high energy techniques, such as microwave or ultrasound, it was possible to enhance the extraction efficiency and reduce the usage of solvents for the extraction of polyphenolic antioxidants from sunflower kernels and hulls [11].

The controversial issue surrounding the benefits of dietary antioxidants for health promotion is the lack of clinical evidence and specific molecular markers able to measure the impact of dietary antioxidants, not only on oxidative stress status, but on health status [12]. For example, vitamin E is known to be a lipid-soluble antioxidant for the prevention of autoxidation of polyunsaturated fatty acids in cell membranes [13]. However, its role in health promotion and disease prevention is not clear. Numerous clinical studies have resulted in conflicting conclusions. One of the reasons for this could be that different forms of “vitamin E” (natural, synthetic, alpha-tocopherol esters etc.) were used in the clinical trials. Moreover, different tocopherols (vitamers) may exhibit different bioactivities. On the other hand, the effects of vitamins on humans are dependent on subtle differences between the genetics of individuals. This is highlighted in the review by Cervantes and Ulatowski on vitamin E and Alzheimer’s disease [14].

Most radical scavengers found in foods are sacrificial antioxidants, which are sensitive to oxidation by reactive oxygen species in the human body. Therefore, the intake of dietary antioxidants should be translated to the reduction of oxidative stress, thus helping to maintain the normal physiological function of human organs. In an overview by Wilson and coworkers [15], the authors examined our understanding of the relationship between brain function and dietary choices, mainly in older humans, and the potential consequence of such choices on health and disease, particularly dementia and Alzheimer’s disease. Health promotion in older persons may be achieved through judicious choice of the frequency and patterns of dietary intake in order to achieve homeostasis or dynamic balance of oxidation and antioxidation. However, the challenge is that there are no straightforward and reliable methods yet to determine the oxidative stress status and antioxidant capacity in human beings. The molecular markers of oxidative stress are exceedingly complex because of the combination of different reactive oxygen species and the biomolecules (lipids, proteins, and DNA/RNA). Although a balanced diet is desirable, there is no way to tell whether a diet is balanced or not, due to the lack of proven biomarkers. The same challenge is faced in the study of the impact of under- and over-nutrition, which are major common challenges in developing countries. This is highlighted by Vassalle and coworkers in their review article, which focuses on case studies of Mauritania and India. It is apparent that the future direction of studies in oxidative stress and dietary antioxidants should focus on mapping out the cause and effect relationship at a molecular marker level of the intake of specific dietary antioxidants, and the output in terms of oxidative stress biomarkers, which could be an indication of risk factors of chronic disease. With the advancement of analytical methods, this goal could be achieved in the near future.
